# Identification of nonlinear-nonlinear neuron models and stimulus decoding

**DOI:** 10.1186/1471-2202-14-S1-P367

**Published:** 2013-07-08

**Authors:** Aurel A Lazar, Yevgeniy B Slutskiy

**Affiliations:** 1 Department of Electrical Engineering, Columbia University, New York, NY, 10027, USA

## 

The majority of neural encoding models employed today consist of a linear feature-selection stage followed by a static memoryless nonlinearity generating the neuronal rate of response. Although such linear-nonlinear (LN) models have been proven useful in characterizing computation in many neurons (see [1] and references therein), they exclude the biophysics of action potential generation and, with a few exceptions [2-4], are limited to describing only linear computations. A large body of evidence [5] suggests that various synaptic microcircuits and complex dendritic morphologies exhibit a rich repertoire of nonlinear computations on a single neuron level. Importantly, such computations are observed not only for individual inputs [2,3] but also between inputs, as manifested by multiplicative interactions between them [5]. At the same time, the highly nonlinear nature of action potential generation has been shown to adversely affect many existing identification methodologies and typically presents itself as artifactual stimulus processing [3,6].

Here we investigate nonlinear-nonlinear neuron models that combine both biophysics and dendritic computation. Specifically, we consider models in which the nonlinear dendritic processing of input stimuli is described by a Volterra series [4] and the aggregate dendritic current is encoded into a sequence of action potentials by a biophysical conductance-based model. We first present a novel experimental approach for characterizing the biophysical spike generator using conditional phase response curves [7]. We then prove that only a projection of Volterra kernels onto the space of input signals can be identified and present an efficient algorithm for estimating these projections. The identification is robust in the presence of noise, scales favorably with the order of nonlinear interactions and affords high-resolution identification due to a basis decomposition of the projections. Furthermore, either synthetic or naturalistic stimuli can be used to identify the model. Finally, we consider a nonlinear-nonlinear population encoding scheme and construct a nonlinear decoder that is capable of reconstructing the original stimulus directly from spike times. This should be contrasted with linear decoders applied to firing rates of LN models [8,9]. To the best of our knowledge, this is the first demonstration of a nonlinear decoder capable of high-fidelity reconstruction from a single multidimensional spike train in the nonlinear-nonlinear setting.
